# Click Chemistry: A Promising Tool for Building Hierarchical Structures

**DOI:** 10.3390/polym14194077

**Published:** 2022-09-28

**Authors:** Adel Badria

**Affiliations:** Department of Fibre and Polymer Technology, KTH Royal Institute of Technology, SE-10044 Stockholm, Sweden; badria@kth.se

**Keywords:** click chemistry, hierarchial structures, 0D/nano/micro building blocks

## Abstract

The hierarchical structures are utilized at different levels in nature. Moreover, a wide spectrum of nature’s properties (e.g., mechanical, physical and biological properties) has been attributed to this hierarchy. Different reviews have been published to cover the use of click chemistry in building hierarchical structures. However, each one of those reviews focused on a narrow area on this topic, i.e., specific chemical reaction, such as in thiol-ene chemistry, or a specific molecule or compound such as polyhedral oligomeric silsesquioxane, or a certain range of hierarchical structures between the nano to micro range, e.g., nanocrystals. In this review, a frame to connect the dots between the different published works has been demonstrated. This article will not attempt to give an exhaustive review of all the published work in the field, instead the potential of click chemistry to build hierarchical structures of different levels using building blocks of different length scales has been shown through two main approaches. The first is a one-step direct formation of 3D micro/macrometer dimensions structures from Pico dimensions structures (molecules, monomers, etc.). The second approach includes several steps Pico ➔ 0D nano ➔ 1D nano ➔ 2D nano ➔ 3D nano/micro/macro dimensions structures. Another purpose of this review article is to connect between (a) the atomic theory, which covers the atoms and molecules in the picometer dimensions (picoscopic chemistry set); (b) “nano-periodic system” model, which covers different nanobuilding blocks in the nanometers range such as nanoparticles, dendrimers, buckyball, etc. which was developed by Tomalia; and (c) the micro/macrometer dimensions level.

## 1. Introduction

Hierarchical structures are an essential part of numerous types of architecture in nature. They are defined as the presence of different structural elements with different length scales in a single body [1]. This different length scale gives each hierarchical structure its “order, n” and characteristic properties [2]. The higher the (n) the more sophisticated hierarchical structures; where n = 0 refers to continuum materials with only a single length scale. Noteworthy, several composites are considered low-ordered hierarchical structures.

In nature, hierarchical structures are usually classified into four main categories [3]; (a) porous hierarchy: in which the pore size distribution in a certain body falls in different length scales: nano, micro, meso and macro. This allows an efficient mass transfer and gas exchange; (b) morphological hierarchy: in which multi layers or shells arranged after each other are utilized. This allows an efficient coupling; (c) structural hierarchy: in which repetitive building blocks are precisely arranged to form these structures. Such arrangements provide a wide range of optimum mechanical properties for the whole body, (d) compositional hierarchy: in which the composition of the building blocks is as important as their structural arrangements.

Hierarchical structures are not only limited to nature. Different reviews have covered the different fabrication methods for the different hierarchical structures; freeze casting [4], layer-by-layer (LBL) assembly [5] and different deposition techniques [6]. Only a few reviews covered the use of click chemistry in building hierarchical structures. Moreover, each one of those reviews focused on a specific narrow area on this topic; some focused on the method for hierarchical structure, e.g., LBL for electronics applications [7], and others covered only specific molecules or compounds such as polyhedral oligomeric silsesquioxane [8], poly(ethylene glycol) (PEG) hydrogels [9].

In this review, a frame to connect the dots between the different published works has been demonstrated. This article will not attempt to give an exhaustive review of all the published work in the field, instead, the potential of click chemistry to build hierarchical structures of different levels using building blocks of different length scales has been shown through two main approaches. The first one is a one-step direct formation of 3D micro/macrometer dimensions structures from Pico dimensions structures (molecules, monomers, etc.). The second approach includes several steps pico ➔ 0D nano ➔ 1D nano ➔ 2D nano ➔ 3D nano/micro/macro dimensions structures.

Another purpose of this review article is to connect between (a) the atomic theory which covers the atoms and molecules in the picometer dimensions (picoscopic chemistry set), and (b) the “nanoscopic chemistry set”, which covers different nanobuilding blocks in the nanometer range, such as nanoparticles, dendrimers, buckyballs, etc., which were developed by Tomalia [10] and (c) the micro/macrometer dimensions level.

## 2. Click Chemistry: History, Definition and Applications

The dawn of modern chemistry dates to the onset of the 18th Century. Since then, chemistry passed through different major stations. One of them was a new synthesis paradigm called click chemistry, or “CC”. This paradigm was proposed by American chemist Karl Sharpless and coworkers, for building a wide range of new chemical compounds using a simple approach.

In general, organic compounds are formed of a backbone of carbon atoms (representing building blocks), linked together to form bigger molecules. In practice, this process of adding carbon atoms to each other is not flawless. Instead, time consumption, expensive costs, low yield, low selectivity of the different reactions used, the complicated purification process required to separate the desired product, the aggressiveness of the used solvents and reactants, the low stability of the final products and the different stereoisomers formed for different reactions are all common drawbacks for the traditional synthesis approaches.

In contrast to the traditional synthesis approaches, click chemistry exploits certain reactions, which are as simple as the clicking of interlocking fasteners hence the name was coined, for forming endless numbers of linkage chemical bonds [11]. Usually, those reactions are selected based on fulfilling certain criteria as shown in Table 1.

**Figure 1:**
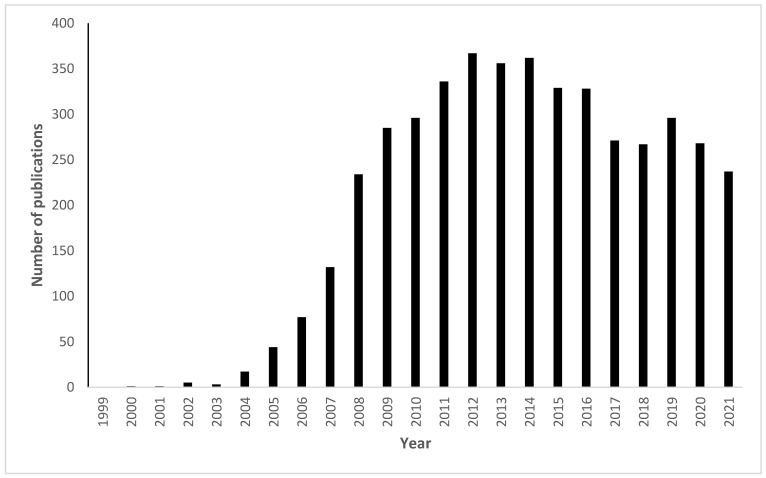
Shows the increase in the number of publications with click chemistry in their titles in the Google Scholar search engine. In the search engine, the following keyword was used “click chemistry” and the function "allintitle:" restricted the search only to the word in the title. Using the “time custom range” function, each year was searched separately, e.g., for the year 2005, the range was adjusted as follows: 2005–2005; the search was conducted on the 12th of September 2022.

Although some of those reactions were already known before the CC terminology was coined, still, the number of publications using the click chemistry term showed a significant increase over the past 20 years as shown in Figure 1.

**Figure 2:**
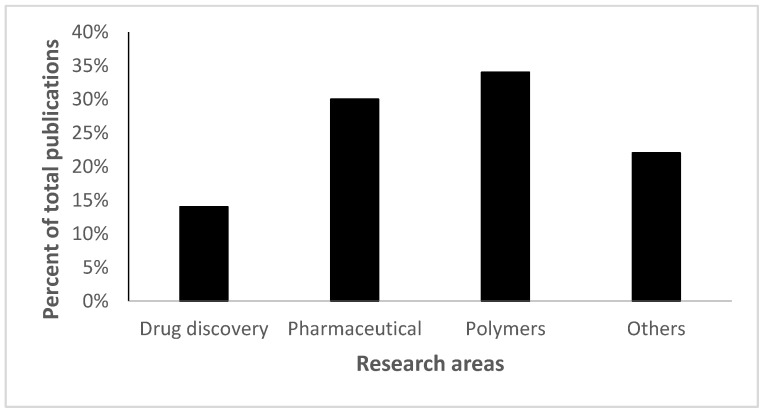
Click chemistry applications fall into four different categories; polymers (34%) represent the highest percentage while drug delivery represents the lowest (14%). The figure has been reproduced from another article [12] under a CC−BY license.

Moreover, this fast expansion of this emerging field was not just limited to the scientific literature, a broad spectrum of applications in different industries showed a similar rise as shown in Figure 2.

**Figure 3:**
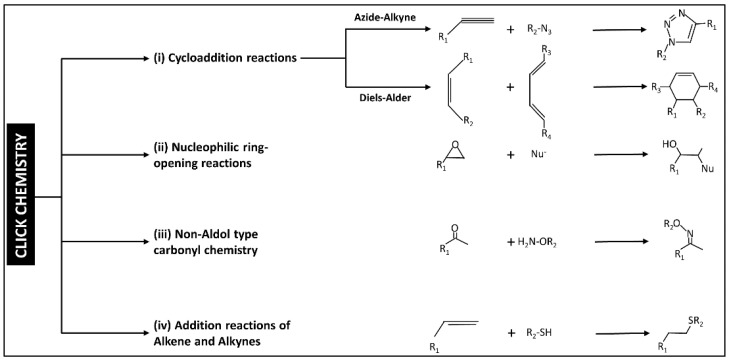
The scheme shows the classification of click chemistry reactions into four main categories. The scheme has been reproduced from another article [13] with permission from Elsevier, 2022.

Historically, the first reaction to being identified as click chemistry is the copper-catalyzed azide-alkyne reaction to form a 5-membered ring. Nowadays, the click chemistry approach is being handled as a toolbox that different groups worldwide are adding to and modifying with different new reactions. So far, four categories have been identified to represent the different click chemistry reactions as shown in Figure 3.

## 3. Hierarchical Structures and Click Chemistry

For any hierarchical structures in nature there have been repeated smaller building blocks which have been arranged in a certain manner to provide the required functionality for this hierarchical construction.

**Figure 4:**
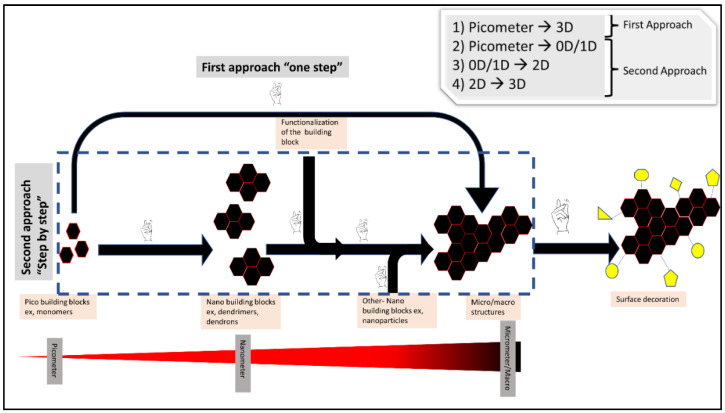
Shows the different possible ways of using click chemistry for building hierarchical structures.

The idea of building blocks for hierarchical structures intersects perfectly with the modularity concept in click chemistry. Click chemistry is a powerful tool for constructing nano, micro and macro structures through two different approaches: (A) the first approach: through direct crosslinking of (pico-building blocks) monomers give a final micro/macro structure such as hydrogels [9,14]; (B) the second approach: through nano-building blocks formation using click chemistry (e.g., dendrimers and dendrons) followed by connecting and crosslinking those formed nano-building blocks again using click chemistry to form bigger structures (Figure 4).

Moreover, click chemistry acts through different pathways for building higher structures either alone or in combination with different assisting chemistries, techniques, and tools. While in the first approach mentioned above, the role of click chemistry involves connecting the different building blocks, using the spatial hindrance and the chemical natures of the involved compounds to give the final topological and internal structures of the final product. In the second approach, click chemistry is used as an assisting tool for the fabrication techniques, such as STAMP-Lithography, SLA 3D printing, phase separation, self-assembly, etc.

Noteworthy, during this review, the following considerations have been taken: 0D is the xyz < 100 nm, 1D xy < 100nm and 2D x or y < 100 nm [15]. Additionally, there has been no differentiation between micro and higher dimensions. Moreover, as molecules, atoms and monomers do not fall into any of those categories, a general term to describe them was used: “Pico building blocks”.

### 3.1. The First Approach

All the micro/macro structures to be constructed must pass first through the nanoscale level. However, what is meant in this category is the whole continuous process (click orthogonal), i.e., from the pico/0D nano to the micro/macro without the involvement of intermediate processing and modifications.

**Figure 5:**
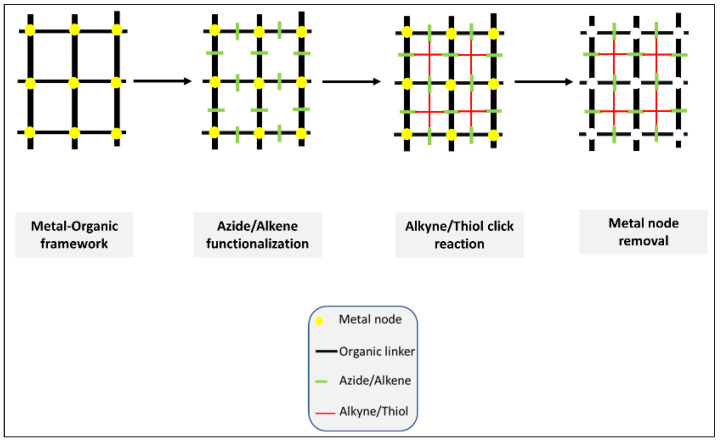
Hierarchical structures from MOF (Metal/covalent Organic frame) using click chemistry. A good example of the first approach. The illustration is based on the work conducted by [16].

In 2020, Hassan et al. published an excellent review article discussing the construction of hierarchical structures from MOF (Metal/covalent Organic frame) using click chemistry (Figure 5) [16]. The significant potential of clickable MOF for building high precision hierarchical structures, porosity control, the transformation of internal and external structures and post-synthesis modifications (PSM) opened a new horizon in the field of bio/material science.

**Figure 6:**
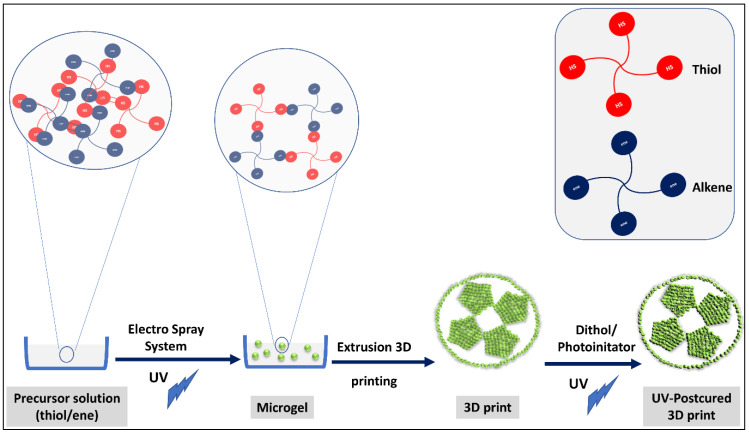
Clickable PEG hydrogel microspheres as building blocks for 3D bioprinting. The illustration is based on the work conducted by [17].

On the other hand, one of the good examples in the literature manifesting this concept of combining click chemistry with 3D printing for building hierarchical structures is the 3D printing of clickable microsphere [17]. Xin et al. clicked norbornene—PEG with excess Dithiol-PEG to give gel microspheres, and the formed microsphere building blocks were extruded using a 3D printer under UV to form a hierarchical structure through S-S bonds between excess added dithiol-PEG (Figure 6).

Among the enormous publications in this area, three examples are demonstrated for the direct formation of micro/macro structures from pico-building blocks: gels, crystals, and controlled porous structures.

#### 3.1.1. Gels

Different forms of gels were shown in literature such as hydrogel and aerogels. **Hydrogels** were prepared using wide variations of clickable monomers or polymers, e.g., Pentaerythritol Propoxylate Tris(3-(furfurylthiol)- propionate) (PPTF) [18], poly(ethylene glycol) diazides [19]. Poly(ethylene glycol) Tetra-azide [20], PEG norbornene and cross-linker dithiothreitol (DTT) [21] using different click chemistries, such as CuCCA [19], SPAAC [20], thiol-ene [21] and Diels-Alder [18] for different applications such as wound healing [22], bone regeneration [23], and heart valve engineering [24]. 

**Aerogel** is another type of gel that was produced directly from a pico-size monomer into a micro/macrostructure. X. Wang et al. prepared inorganic aerogel in which thiol-ene click chemistry is used to prepare ZrO_2_-SiO_2_ aerogel composite [25]. The prepared aerogel showed better thermal stability over the pristine ZrO_2_.

Also, an **organogel** has been synthesized using a Mechano-activated click chemistry to mimic biological materials such as bone and muscles. In which a linear polytriazole was formed using ultrasound-activated CuAAC click chemistry. The novelty in this work lies in the usage of polymer-destructive energy, e.g., ultrasonic to construct a stable gel through ultrasonic-activated click chemistry [26].

#### 3.1.2. Crystals

The ability of click chemistry to control the formed crystal structures packing (BCC, FCC, etc.), the porosity and the topological features have been shown in the process of forming different crystals.

Zhang et al. reported the use of BPOSS-C60 0D building blocks for constructing micro/macro-sized solid **crystals**. BPOSS-C60 0D building blocks were crosslinked using click chemistry to give micro/macrocrystals in different packing levels (BCC, FCC) [27].

Another type of crystal, **liquid crystals**, have been synthesized using a natural product with high biological activity “[1–3]-Triazoles”. The use of Huisgen Cu(I)-catalyzed cycloaddition reaction provided a promising way for producing the triazoles with specific biologically valuable crystallographic phases [28].

#### 3.1.3. Controlled Porous Structures

The combination of click chemistry, multiporous polymers and Breath techniques provides a precise way for the formation of microfilms with well-controlled shape and size pores. Mongkhontreerat et al. used multipurpose linear-dendritic block copolymers (MLDBC) to produce membranes with controlled porosities. The fine-tuning between those different (MLDBC) would provide a wide control over the membrane pores number, size and other surface properties [29].

At the same time, the use of orthogonal click chemistry with LBL assembly allowed for an efficient control of microporosity and wettability of the fabricated nanofilm [30]. The procedure basically combines thiol–yne click chemistry with lithographic techniques (mask) to produce such structures.

Another example is the SiO_2_ monolith, which was prepared from vinyl-POSS and dithiol linkers prepared in quartz glass tubes using PRTEA (photochemical radical thiol-ene addition) [31].

### 3.2. The Second Approach

#### 3.2.1. Picometer ➔ 0D-Nanometer

For such small dimensions of controlled fabrication, a significant number of articles and review articles have already been published covering the usage of click chemistry to synthesize 0D structures: dendrimers, dendrons, hyperbranched polymer [32], single chain nanoparticles [33] and sequence-controlled polymers [34].

Other reactions which do not include a construction frame behind it, e.g., conjugation for labelling or targeting purposes have also been shown in literature such as nanoparticles [35], buckyballs [36], micelles [37] and microparticles [38].

#### 3.2.2. 0D/1D-Nanometer ➔ 2D-Nanometer

**Figure 7:**
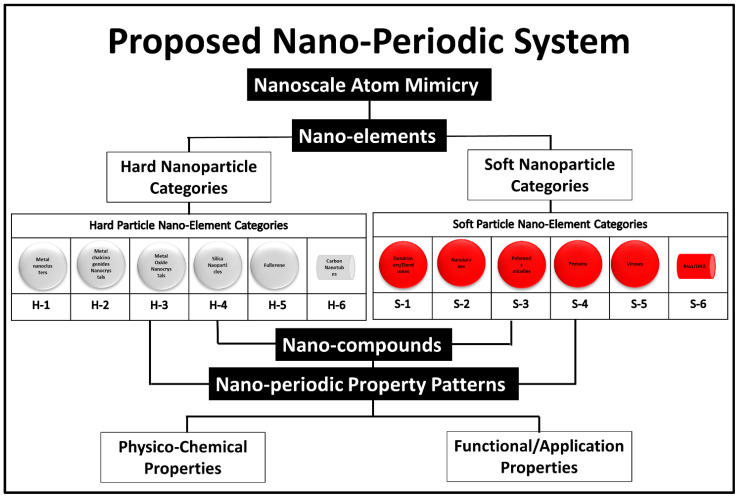
Proposed nano system by Donald Tomalia. The figure has been reproduced from another article [10] under a CC−BY license.

The idea of creating a unified theory covering the use of 0D-nanobuilding blocks for building higher structures was a detailed cornerstone theoretical work of Tomalia et al. 2009 [10]. In his published works, Tomalia suggested a similar system to the periodic atomic table system for higher structures (0D) [39]. The dendrimers were used as the explanatory model for his idea. The 0D nanoscale building blocks were categorized into two main classes, hard and soft nanostructures, and under each category six subcategories were proposed based on compositional/architectural considerations as shown in the following (Figure 7).

This model is similar to the atomic theory in many different aspects, e.g., the concept of quantization, the Aufbau principle, the number of surface groups (vs. number of electrons in atoms) and the MW of the generation (vs. MW of atoms).

Different published work showed further successful details for building hierarchical structures from 0D building blocks. Using nanobuilding blocks, Rudick and Percec in their work on dendrimers and dendrons showed a high precision control of the final structures depending on the generations used [40]. The lower the generations, the lesser the branching and hence the steric hindrance between different branches leading to the final geometrical structures of more flat structures and vice versa.

Similarly, Zhang et al. studied different functionalized 0D building blocks. In which those nano-building blocks functionalized with click chemistry with oligomer tethers were used [41]. The periodic change in the size, type, and number of those tethers allowed fine control over the final structure.

The use of click chemistry to link different soft/soft, soft hard and hard/hard nano building blocks have been widely recognized. Three different examples will be discussed below to demonstrate those three possible combinations.

##### Soft/Soft Nano Building Blocks

**Figure 8:**
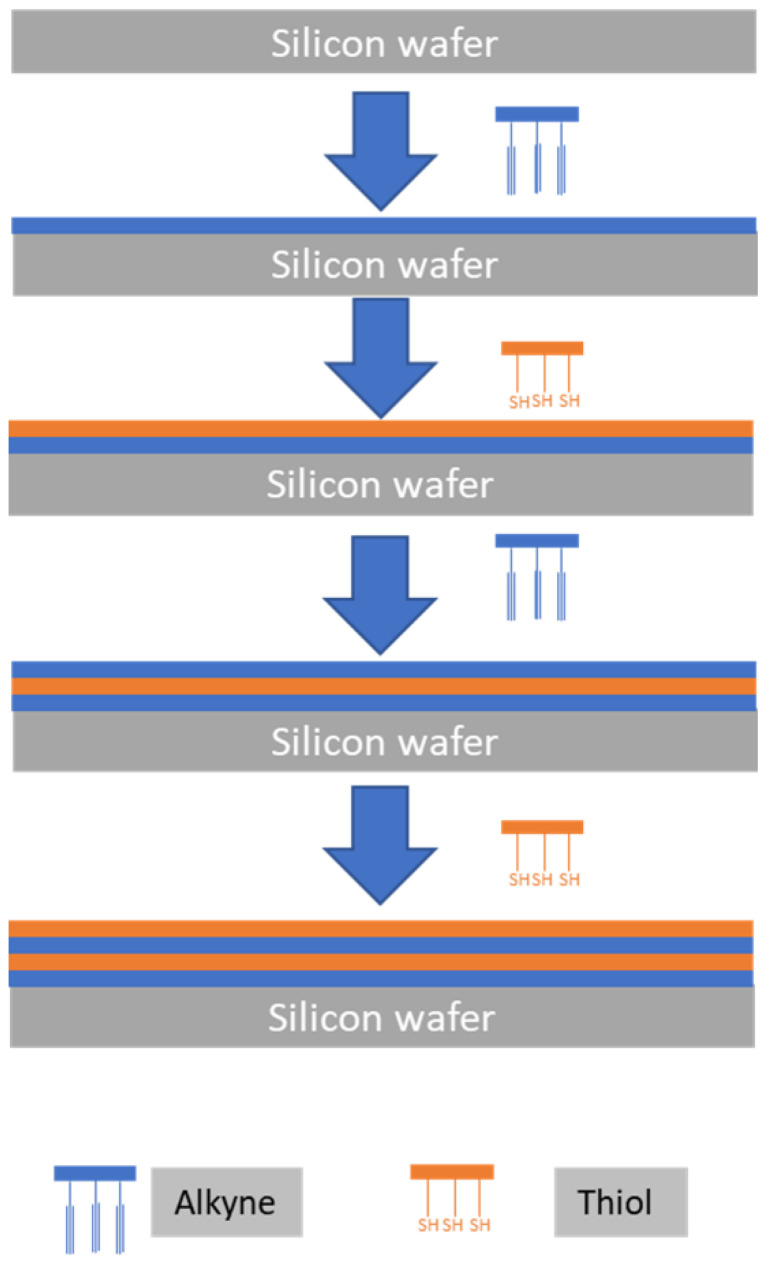
The use of soft/soft building blocks to tailor ultrathin multilayers though click chemistry. The silicon wafer is alternatively coated with azide-functionalized dendrimers and alkyne-functionalized dendrimers. The illustration is based on the work conducted by [42].

Clicking bisMPA based dendrimers/dendrimers, Vestberg et al. 2007, showed a significant control over the hierarchical architecture building up. Using CuCCA click chemistry, the film thickness could be accurately controlled by manipulating the dendrimers size (generation) and layer thickness [42] (Figure 8). The exploitation of dendrimers from different materials whether alone or in combination, core sizes and branch lengths would open endless possibilities for the control of different layers thickness.

While the construction of hierarchical structures of dendrimers/dendrimers using click chemistry is relatively common [43,44,45], the other pairs of soft nanobuilding blocks hierarchical fabrication using click chemistry, such as dendrimers/ RNA [46], dendrimers/DNA [47], micelles/ dendrimers [48], etc., are more restricted to labelling, drug and gene delivery application rather than being used for hierarchical structures. On the other hand, an excellent review covering these pairs’ potential to form hierarchical structures using other chemistries has been published elsewhere [49].

##### Hard/Hard Nano Building Blocks

For electro/photocatalytic applications, alkyne-azide click chemistry showed a very efficient potential to form mono/multilayers, hetero/homo structure on different substrates using metallic, semiconductor and dielectric nanoparticles. Upadhyay et al. showed even further control over the packing ratio and crystal unit by controlling the number of click groups on the substrates, substrate surface roughness and the solvent used [50]. Williams and Teplyakov showed another metal/metal assembly, in which azide-terminated Si nanoparticles and alkyne-terminated Si nanoparticles were added alternatively to a gold substrate using click chemistry to form nearly 100% coverage of the substrate [51]. For self-healing polymers applications, Le Neindre and Nicolaÿ, used a thiolated copolymer with silver nanoparticles as crosslinkers [52]. The self-healing potential was attributed to the reversible thiolates exchange that takes place over the Ag nanoparticles.

**Figure 9:**
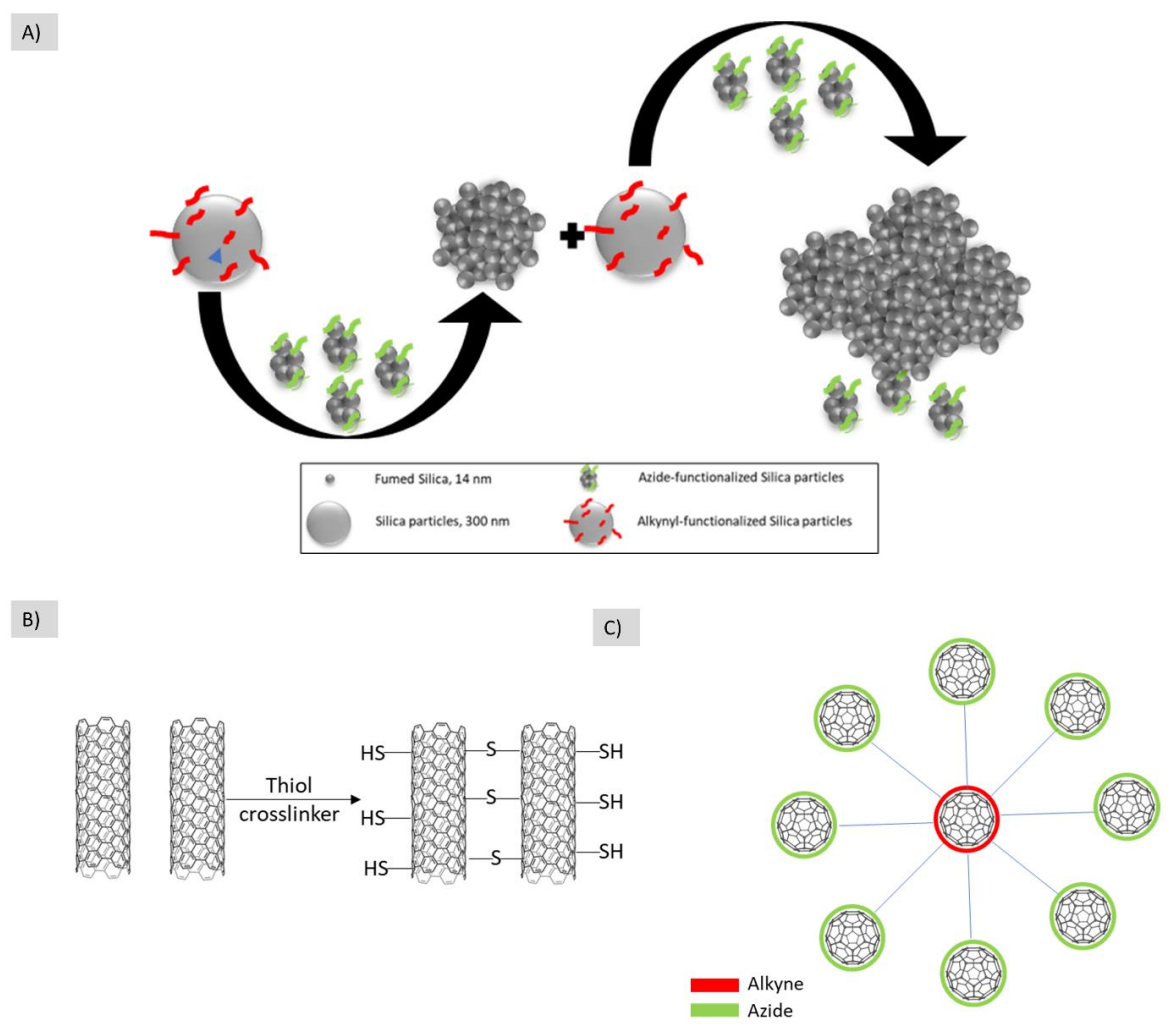
Different examples for the use of hard/hard nanobuilding blocks for building hierarchical structures. (**A**) Azide-funtionalized fumed silica is reacted with alkyl-functionalized silica particles to form hierarchical structures, (**B**) nanotubes are assembled using thiol crosslinkers after reacting with the ene in the nanotube structure, (**C**) the core fullerene is functionalized with azide while the shell ones are functionalized with alkyne to form this hierarchical structure. The illustrations are based on the work conducted by [53,54,55], respectively.

Not only metallic nanoparticles were able to form hierarchical structures, non-metallic azide-functionalized silica particles were also clicked with Alkyne functionalized-silica particles to form the building block aggregates. The further repetition of this step resulted in building a hierarchical structure [53] (Figure 9A).

In addition to the nanoparticles, in the same category of hard/hard nanobuilding blocks lies the carbon/carbon nanotube [55] (Figure 9B) and Fullerene/Fullerene clicking [54] (Figure 9C).

##### Soft/Hard Nano Building Blocks

**Figure 10:**
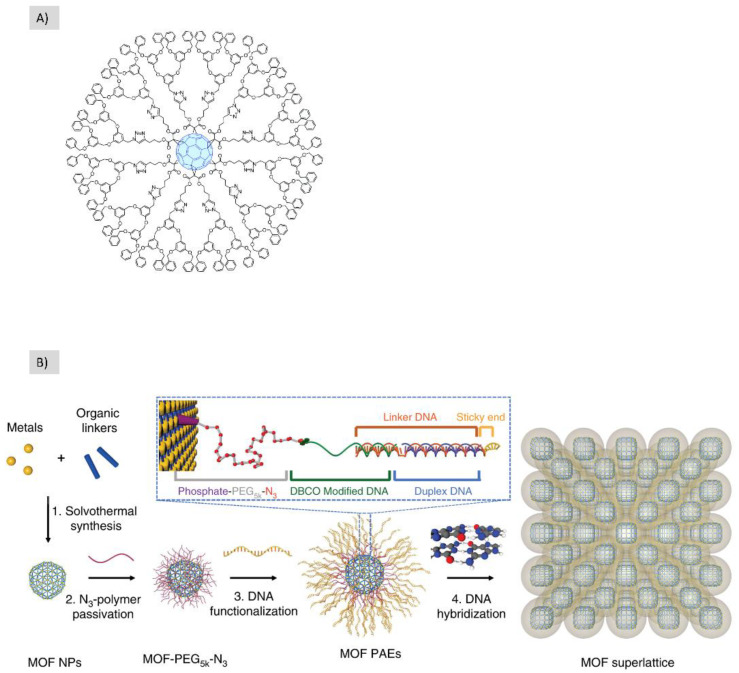
The use of soft/hard nanobuiling blocks to fabricate hierarchical structures using click chemistry. (**A**) Fullerene is used as the core for the formation of this dendrimer, (**B**) the polymeric nanoparticles and DNA are coupled using an azide/alkyne click reaction followed by DNA hybridization to form the final hierarchical structure. These figures have been reproduced from other articles [54,58] under a CC−BY license.

Fullerene and dendrimers represent different types of hard and soft building blocks, respectively. The clicking between those two types both dendrimers in the core and fullerene in the shells or the opposites has been published in the literature [56,57]. While Hahn et al. 2012 used fullerene as a peripheral decoration of the polypropylene simine (PPI) dendrimers, Nierengarten 2017, showed different dendrimers using fullerene hexa-adduct core building blocks bearing twelve equivalent clickable groups (Figure 10A). Their post-modification allows for the introduction of twelve equivalent peripheral subunits.

The clicking between DNA and nanoparticles represents another example of hard/soft building blocks clicking. S. Wang et al. showed the ability to build hierarchical structures through Cu-free click chemistry between MOF nanoparticles and DNA which were then programmed into superlattice organization [58] (Figure 10B).

#### 3.2.3. 2D-Nanometer ➔ 3D

In contrast to the other shown categories, the 2D ➔ 3D fabrication using click chemistry is not significantly mentioned in the literature. Moreover, several comprehensive reviews such as [59,60,61,62] in the field did not mention click chemistry at all as a possible method for such a purpose. However, a few examples found in the literature are reviewed in this section.

The first example is the use of molybdenum disulfide MoS_2_ nanoflakes in the formation of a hydrogel composite using N,N′-methylenebis(acrylamide) [63]. The formed composite has the potential to self-heal at 70 °C which allows it to be an efficient building block for bigger structures. Similarly, exfoliated MoS_2_ nanosheets reacted with chitosan-treated polystyrene spheres to form a hierarchical core/shell structure to improve thermal stability and fire safety [64].

**Figure 11:**
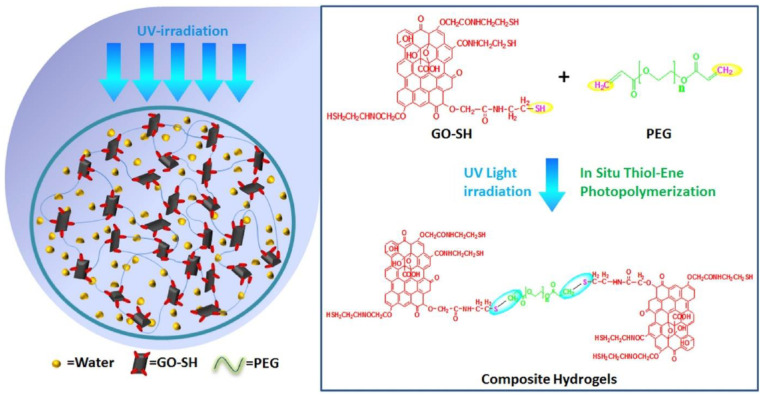
An example of the use of 2D nanosheets to construct a 3D hierarchical porous structure using click chemistry. The thiolated graphene oxide reacted with the PEG through thiol-ene chemistry. This figure has been reproduced from another article [66] with permission from Elsevier, 2022.

Graphene oxide is a 2D structure which has been frequently functionalized using click chemistry [65]. In the context of 2D ➔ 3D fabrication, a thiolated graphene oxide reaction with polyethylene glycol has been published [66]. Such a thiol-ene reaction allowed for the formation of a hydrogel with hierarchical porosity which showed an extremely efficient potential in the field of wastewater treatment (Figure 11) [66].

In the last example, 2D nanocrystals of cellulose were assembled to form a 3D nanoplatelets gel [67]. This was achieved through the functionalization of two sets of cellulose nanocrystals; one with azide and the other one with alkyne then the Cu(I)-catalyzed Huisgen 1,3-dipolar cycloaddition click reaction was allowed to take place.

## 4. Conclusions

Different techniques, materials and chemistries have emerged to fulfil the need for the construction of hierarchical structures. One of the most promising chemistries for such a purpose is click chemistry, where it introduces a simple way to obtain the desired compounds efficiently. The mild conditions needed for such chemistry are its simplicity, stable reactions and results allowed for a significant tool for building hierarchical structures to be created.

In this review, the different approaches for building different hierarchical structures using click chemistry through two different main approaches have been presented. Another aim of this review is to provide a framework that connects the atomic theory, the nanoscale system proposed by Tomalia and the micro/macro dimension systems. This task was quite challenging on some length scale fabrication levels, e.g., 2D nano ➔ 3D and 0D nano ➔ 2D soft/soft nanoparticles. The possible reason is that the nanoscale system is similar to the atomic system in the need for diverse bonding systems, i.e., the atomic system utilizes strong bonds (ionic, covalent, metallic and molecular) and weak bonds (Hydrogen, dipole-dipole, Van der Waals, etc.). Likewise, the use of only one set of chemical reactions (click chemistry) seemed challenging to connect all the different levels of hierarchical structures discussed in this review. Further comprehensive reviews to cover the different chemistries involved to build hierarchical structures in this frame (atomic-nano-micro/macro structures) would be a unique addition to the field.

## Figures and Tables

**Table 1:** Criteria for defining a certain reaction as a click reaction.

Advantage	Explanation
**High Selectivity**	-
**High yield**	-
**Mild conditions**	Usage of mild solvents and reactants
**Fast reaction**	The exothermicity of both thermodynamic and kinetics of the reaction allowed fast and stable final products
**Minimum byproducts**	-
**Easy purification**	No need for complicated chromatography or other purification techniques
**Modularity**	The ability of forming diverse library of chemical structures from simple chemical modules
**Regiospecific**	Minimum stereoisomers are being formed
